# Increased Risk of Traumatic Injuries among Parents of Children with Attention Deficit/Hyperactivity Disorder: A Nationwide Population-Based Study

**DOI:** 10.3390/ijerph18073586

**Published:** 2021-03-30

**Authors:** Dian-Jeng Li, Yi-Lung Chen, Ying-Yeh Chen, Ray C. Hsiao, Wei-Hsin Lu, Cheng-Fang Yen

**Affiliations:** 1 Department of Addiction Science, Kaohsiung Municipal Kai-Syuan Psychiatric Hospital, Kaohsiung 80276, Taiwan; u108800004@kmu.edu.tw; 2 Department of Nursing, Meiho University, Pingtung 91200, Taiwan; 3 Graduate Institute of Medicine and School of Medicine, College of Medicine, Kaohsiung Medical University, Kaohsiung 80708, Taiwan; 4 Department of Psychology, Asia University, Taichung 41354, Taiwan; elong@asia.edu.tw; 5 Department of Healthcare Administration, Asia University, Taichung 41354, Taiwan; 6 Taipei City Psychiatric Center, Taipei City Hospital, Taipei City 107, Taiwan; ychen@tpech.gov.tw; 7 Institute of Public Health, School of Medicine, National Yang-Ming University, Taipei City 112, Taiwan; 8 Department of Psychiatry and Behavioral Sciences, University of Washington School of Medicine, Seattle, WA 98105, USA; rhsiao@u.washington.edu; 9 Department of Psychiatry, Children’s Hospital and Regional Medical Center, Seattle, WA 98105, USA; 10 Department of Psychiatry, Ditmanson Medical Foundation Chia-Yi Christian Hospital, Chia-Yi City 60002, Taiwan; 11 Department of Psychiatry, Kaohsiung Medical University Hospital, Kaohsiung Medical University, Kaohsiung 80708, Taiwan

**Keywords:** attention-deficit/hyperactivity disorder, parents, children, traumatic injuries

## Abstract

Children with attention deficit/hyperactivity disorder (ADHD) are vulnerable to traumatic injuries. Parents of children with ADHD experience undesirable impacts more frequently than parents of children without ADHD. The aim of this study was to evaluate whether traumatic injuries are more prevalent in parents of children with ADHD than in parents of children without ADHD. We compared the prevalence of traumatic injuries between parents of children with and without ADHD by using data from the Taiwan Maternal and Child Health Database from 2004 to 2017. The Cox proportional-hazards regression model was used to examine differences in burn injury, fracture, and traumatic brain injury between parents of children with and without ADHD after adjustment for age, urbanicity, and income level. In total, 81,401 fathers and 87,549 mothers who had at least one offspring with ADHD and 1,646,100 fathers and 1,730,941 mothers with no offspring with ADHD were included in the analysis. The results indicated that both fathers and mothers of children with ADHD had higher risks of burn injury, fracture, and traumatic brain injury than fathers and mothers of children without ADHD. Mothers of children with ADHD had higher risks for all kinds of traumatic events than fathers of children with ADHD.

## 1. Introduction

### 1.1. Attention-Deficit/Hyperactivity Disorder and Risk for Injury

Attention-deficit/hyperactivity disorder (ADHD) is the most common neurodevelopmental disorder characterized by inattentiveness, hyperactivity, and impulsivity [1,2]. The prevalence of ADHD is estimated at approximately 5–7.2% in children [3,4] and 2.5% in adults [5] globally. The economic burden of ADHD ranged from US$143 to 266 million in the United States [6]. Studies have identified strong links between ADHD and adverse life outcomes [7,8]. Children with ADHD were shown to be more likely to have poor relationships with peers [9] and family [10], worsened quality of life [11], impaired academic performance [12], and even higher mortality [13] than those without ADHD.

Compared with healthy peers, children with ADHD are more prone to accidental injury and trauma [14,15], which may lead to increased mortality [16]. Hyperactivity, impulsiveness, and inattentiveness may all contribute to injury [17]. A nationwide population-based study in Taiwan revealed that unaffected siblings are also more prone to traumatic brain injury than the control group [18]. The core symptoms of ADHD (inattention, hyperactivity, and impulsivity) shared by children and adolescents with ADHD and their siblings have been linked to a higher risk of accidental injury and trauma [18,19,20]. Such incidence of injury could be reversed under adequate treatment of methylphenidate [21]. In addition, parents’ adapting behavior also influenced their children’s behavior. A comprehensive meta-analysis, which partially included studies regarding children with ADHD, reported that unintentional injuries in children could be reduced by intervention to parenting skills, such as parenting education and home-visiting programs [22].

### 1.2. Do Parents of Children with ADHD Have a Higher Risk of Traumatic Injuries than Parents of Children without ADHD?

Although the association between ADHD and traumatic injuries in children had been explicated, the impact of children’s ADHD on their parents warrants further study. A review of twin studies revealed that the heritability of ADHD ranges from 77% to 88% [23]; however, studies on the heredity of ADHD between parents and children have been scarce [24,25]. Several factors hinder investigations on the diagnosis of ADHD among parents of children with ADHD, including parents’ recall bias, confounding by comorbidity (mood disorder or substance abuse), and underdiagnosis of adult ADHD [26]. The results of studies assessing co-segregation of endophenotypes, including executive function and attention-deficit from parents to children are contrasting [27,28]. Examining the risk of traumatic injuries in parents of children with ADHD may provide more information on the parent–child transmission of ADHD.

Parents of children with ADHD experience undesirable impacts more frequently than parents of children without ADHD. In comparison with parents of children without ADHD, parents of children with ADHD reported lower self-efficacy to help their children, felt less welcome and supported by their children’s schools and teachers, and perceived less time and energy for involvement in their children’s academic lives and social interaction [29]. Families with children affected by ADHD experienced greater economic burden than did families without children affected by ADHD [30]. Moreover, parents of children with ADHD had increased psychological stress, and the care burden was significantly associated with psychological symptoms [31]. Given the biological and psychological factors mentioned above, it is reasonable to hypothesize that the parents of children with ADHD may be more vulnerable to traumatic injuries than those who had no children with ADHD.

### 1.3. Aim of The Study

We used the Taiwan Maternal and Child Health Database (TMCHD) to investigate the risk of traumatic injuries in parents of children with ADHD. This study examined the risk of traumatic injuries in parents of children with ADHD. The results of this study may provide knowledge to develop service programs to enhance parental health. We hypothesized that parents who had children with ADHD were more likely to experience traumatic injuries than those who had no children with ADHD.

## 2. Methods

### 2.1. Population

Our study consisted of liveborn children with complete information about the gestational age at birth and identity of both parents in the TMCHD from 2004 to 2017. The data include 99.78% of all births in Taiwan since 2004 [32]. TMCHD data were retrieved from the Taiwan Birth Registration Database, Birth Certificate Application, National Register of Death, and Taiwan National Health Insurance Research Database. The inclusion criterion of this study is the liveborn children and their parents who were recorded in the TMCHD. The exclusion criterion is parental diagnosis of ADHD; if the parents had a diagnosis of ADHD, data for parent–child pairings were excluded. Because we only focused on the diagnosis of ADHD, only parental ADHD but no other neurological or psychiatric disorders in children or parents was listed in the exclusion criterion. Thus, the children or parents in the current study may be comorbid with other neurological or psychiatric disorders. This study was approved by the Research Ethics Committee of the China Medical University & Hospital (approval number: CMUH108-REC1-142).

### 2.2. Measure

#### 2.2.1. Exposure

In this study, the children’s ADHD status was the exposure. If the children received more than three outpatient diagnoses of ADHD (*International Classification of Disease*, 9th revision, Clinical Modification [ICD-9-CM] code: 314 and 10th revision [ICD-10] code: F90) or one inpatient diagnosis of ADHD within the study period (from 1 January 2004, to 31 December 2017), they were identified as having a diagnosis of ADHD.

#### 2.2.2. Outcome

The main outcome included parental traumatic injuries, including burn injury, fracture, and traumatic brain injury, according to ICD-9 and ICD-10 (Supplementary Table S1). Parental traumatic injuries were not mutually exclusive. Parents were followed up from 1 January 2004, to the event of interest, death, or the end of study.

#### 2.2.3. Covariates

The covariates in this study were parental sociodemographic characteristics comprising individual’s age (the difference between birthdate and the end of study, 31 December 2017), urbanicity (urban vs. rural), and low-income level. The urbanicity was stratified into seven levels from 1 (urbanized cities) to 7 (remote rural areas) based on the development level of each area [33]. We categorized areas ranging between 1 and 3 as urban and between 4 and 7 as rural. Data for low-income households were obtained from the Low-Income and Middle-Low-Income Households dataset, which is managed by the Department of Social Assistance and Social Work, Ministry of Health and Welfare, Taiwan, and contains information about low-income and middle-low-income households since 1 July 2012.

### 2.3. Statistical Analyses

For descriptive statistics, frequency with proportion and mean with standard deviation (SD) are reported for categorical variables and continuous variables, respectively. To determine the risk of traumatic injuries in parents whose children did and did not have the diagnosis of ADHD, we used the Cox proportional-hazards regression model with time-on-study as the time scale, with adjustment for age, urbanicity, low-income level, and competing risk of death. Hazard ratios (HRs) with 95% confidence intervals (CIs) were used to quantify the risk and statistical significance of traumatic injuries, respectively. Parents of children without ADHD served as the reference. An HR value greater than 1 suggested that the risk of traumatic injuries is higher in parents of children with ADHD compared with parents of children without ADHD. If the CI of the HR included the null value of 1, there was no significant difference in the risk of traumatic injuries between parents of children with and without ADHD. We further compared the risks of traumatic injuries between fathers and mothers using the difference of two hazard ratios divided by the square root of the sum of the two squared standard errors [34].

## 3. Results

**Figure 1:**
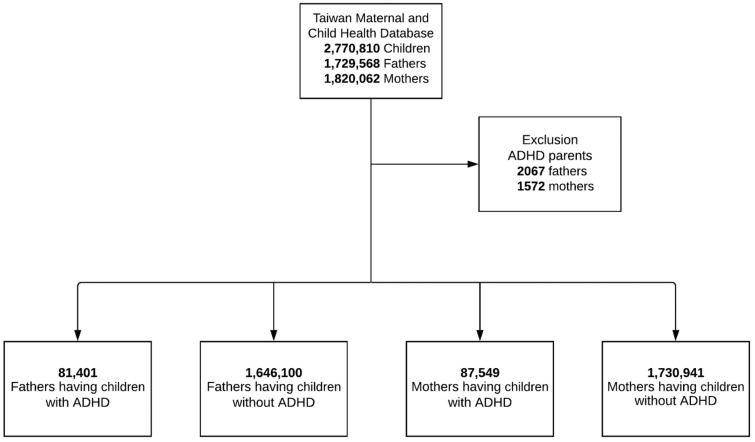
Flowchart of the Study Design.

Figure 1 presents the flowchart of sampling. The target sample included 2,770,810 children born between 2004 and 2017 and their parents. We excluded 3639 parents (2067 fathers and 1572 mothers) ADHD. As a result, we recruited 1,727,501 distinct fathers and 1,818,490 distinct mothers in the study. Of these, 81,401 (4.7%) fathers and 87,549 (4.8%) mothers had at least one child with ADHD, and 1,646,100 (95.3%) fathers and 1,730,941 (95.2%) mothers had no child with ADHD (Figure 1). The characteristics of parents having children with and without ADHD are presented in Table 1. The mean age of fathers and mothers of children with ADHD was 42.9 years (SD = 6.1 years) and 38.9 years (SD = 5.1 years), respectively; the mean age of fathers and mothers of children without ADHD was 40.4 years (SD = 6.9 years) and 37.5 years (SD = 5.7 years), respectively. Most parents lived in urban areas, and a small proportion belonged to the low-income group. The proportions of traumatic injuries for fathers of children with and without ADHD were as follows: burn injury (3.9% vs. 3.5%; χ^2^ = 39.29, *p* < 0.001), fracture (16.5% vs. 15.1%; χ^2^ = 130.34, *p* < 0.001), and traumatic brain injury (5.8% vs. 5.4%; χ^2^ = 24.71, *p* < 0.001). For mothers of children with and without ADHD, the proportions of traumatic injuries were as follows: burn injury (4.6% vs. 3.5%; χ^2^ = 287.87, *p* < 0.001), fracture (9.2% vs. 7.5%; χ^2^ = 354.20, *p* < 0.001), and traumatic brain injury (5.0% vs. 3.9%; χ^2^ = 255.79, *p* < 0.001).

Table 2 presents a summary of the risks of traumatic injuries between parents of children with and without ADHD using the Cox proportional-hazard regression model. Our analyses revealed that even when the parents did not themselves have diagnosed ADHD, they had a higher risk of traumatic injuries if they were parents of children with ADHD than parents of children without ADHD, after adjustment of sociodemographic factors. The risks for traumatic injuries, including crude HRs and adjusted HRs, ranged from 1.08 to 1.45, and tended to be larger after controlling for sociodemographic data. Generally, the increased risks for traumatic injuries were robust and pervasive because they were observed for both fathers and mothers and for various types of traumatic injuries, including burn injury, fracture, and traumatic brain injury. A comparison of the adjusted HRs between the fathers and mothers of children with ADHD revealed that the mothers had greater HRs than the fathers for all types of traumatic injuries (*p* < 0.001).

## 4. Discussion

### 4.1. Main Findings of the Current Study

The results of the current study confirmed the hypothesis that parents of children with ADHD were more likely to experience traumatic injuries than those of children without ADHD. In addition, mothers had greater risks of traumatic injuries than fathers when having children with ADHD. To our knowledge, this is the first study to examine the risks of traumatic injuries for both fathers and mothers of children with ADHD worldwide. The fathers and mothers of children with ADHD had higher risks of burns, fractures, and traumatic brain injury than those of children without ADHD. The current study benefited from the establishment of the healthcare database in Taiwan. The pairs of data for parents and children were derived from the TMCHD [32], which was collected from several databases of public health, such as Taiwan’s National Health Insurance Research Database (NHIRD) [35]. Using the TMCHD, several topics could be researched, including family interaction or heritable disease, in large populations

Several reasons may account for the higher risk of traumatic injuries in parents of children with ADHD. First, ADHD is a highly heritable neurodevelopmental disorder. A review revealed that the estimated heritability of ADHD is as high as 71–90% [36]. A behavioral study of parents having children with ADHD during training programs revealed that parents who did not meet the criteria for ADHD also exhibited moderate symptoms of ADHD, measured using the ADHD Self-Rating Scale [37]. Hence, parents who do not have a formal diagnosis of ADHD but have children with ADHD may present symptoms of hyperactivity, impulsivity, or inattention [17], which may be associated with traumatic injuries.

Second, the undiagnosed ADHD in adulthood may increase the risk of traumatic injuries in parents. Several factors contribute to the underdiagnosis. First, medical record keeping started in 2004 at the TMCHD [32] and parents may have been diagnosed with ADHD before 2004. Second, based on data from the NHIRD, the proportion of youths who received treatment for ADHD increased from 0.11% in 2000 to 1.24% in 2011, indicating a trend of increasing awareness of ADHD and willingness to receive psychiatric treatment [38]. Moreover, it is possible that patients who meet the criteria for ADHD were not identified by their parents and teachers for treatment in their early years. Additionally, ADHD is comorbid with other psychiatric disorders, including substance use disorder [39] and bipolar disorder [40], which may increase the risk of traumatic injuries among parents with undiagnosed ADHD.

Third, although little evidence exists on children with ADHD being violent toward their parents, studies have shown that adults with a history of ADHD are more likely to perpetrate violence to intimate partners than controls [41,42]. Hyperactivity, the core ADHD symptom, was shown to be a significant indicator of domestic violence [43]. Such aggressive behaviors in children with ADHD may be possible leading to traumatic injuries in their parents.

Last, studies have revealed that a high proportion of parents having children with ADHD experience depression [44] and anxiety [45]. Stress-related negative emotions (depression and anxiety) were shown to cause loss of concentration [46], thereby increasing the likelihood of accidental events such as traffic accidents [47] and contributing to the increased risks of traumatic injury among parents of children with ADHD. On the other hand, our findings indicate that mothers are at a higher risk for all types of traumatic events than fathers. Mothers are often the caregivers for children in Taiwan, and a diagnosis of ADHD for children was reported to be a predictor of increased caregiver burden [48].

### 4.2. Limitations

Several limitations of the study must be addressed. First, as a retrospective cohort study, the possibility of missing records for detailed clinical variables limited the presentation of outcome. Second, for a naturalistic observation, confounding factors cannot be controlled to the same extent as that in a clinical trial. However, naturalistic studies could include individuals more representative of real-world clinical settings. Third, individuals included had undergone treatment so that they could be identified in the TMCHD; however, those who did not seek medical help for ADHD or traumatic injuries were not included into the study.

## 5. Conclusions

This study revealed that parents who had at least one child with ADHD were at a higher risk of burn injuries, fractures, and traumatic brain injury compared with those without a child affected by ADHD; moreover, mothers were at a higher risk than fathers for all types of traumatic events. The current study implicates the importance to be aware of the increased risks of traumatic injury among parents of children with ADHD. Proper education about accident prevention and management may be helpful to the parents of children with ADHD. Given that knowledge is fundamental to act, it is essential to educate the parents of children with ADHD the increased risk of traumatic injuries they may have. It is also to help the parents to look back upon their life experiences and detect the histories of encountering traumatic injuries. Detecting the association of inattention, impulsivity, and psychological burden on parenting with the occurrences of traumatic injuries should help the parents to raise alertness of and develop prevention strategies for the possibility of traumatic injuries. The need for behavioral and pharmacological intervention in parents of children with ADHD should be evaluated, especially in the parents with undiagnosed ADHD or subthreshold ADHD symptoms. It deserves further prospective studies with longer follow-up period to explore whether undiagnosed ADHD, care burden of parents, and children’s aggressive behaviors contribute to the increased risks of traumatic injuries in parents of children with ADHD.

## Figures and Tables

**Table 1:** Demographics and traumatic injuries in fathers and mothers of children with and without ADHD.

Variable	Fathers of Children with ADHD	Fathers of Children without ADHD	Mothers of Children with ADHD	Mothers of Children without ADHD
	*N* = 81,401	*N* = 1,646,100	*N =* 87,549	*N* = 1,730,941
Age, Mean (SD)	42.9 (6.1)	40.4 (6.9)	38.9 (5.1)	37.5 (5.7)
Urbanicity, *n* (%)				
Urban	66,073 (81.2)	1,276,763.0 (77.6)	70,979 (81.1)	1,346,539 (77.8)
Rural	15,328 (18.8)	369,337.0 (22.4)	16,570 (18.9)	384,402 (22.2)
Low-income, *n* (%)	4222 (5.2)	51,671.0 (3.1)	4182 (4.8)	48,560 (2.8)
Traumatic injuries, *n* (%)				
Burn injury	3189 (3.9)	57,694 (3.5)	4004 (4.6)	60,365 (3.5)
Fracture	13,451 (16.5)	247,982 (15.1)	8047 (9.2)	129,281 (7.5)
Traumatic brain injury	4721 (5.8)	88,873 (5.4)	4396 (5.0)	68,146 (3.9)

**Table 2:** Risks of traumatic injuries for parents of children with and without ADHD.

Traumatic Injuries	Fathers of Children with ADHD vs. Fathers of Children without ADHD		Mothers of Children with ADHD vs. Mothers of Children without ADHD		Parents of Children with ADHD vs. Parents of Children without ADHD	
	Crude HR (95% CI)	Adjusted HR (95% CI) ^a^	Crude H (95% CI)	Adjusted HR (95% CI) ^a^	Crude HR (95% CI)	Adjusted HR (95% CI) ^b^
Burn injury	1.19 (1.45–1.23)	1.23 (1.19–1.27)	1.33 (1.30–1.36)	1.35 (1.32–1.38)	1.24 (1.22–1.26)	1.30 (1.27–1.32)
Fracture	1.11 (1.09–1.13)	1.16 (1.14–1.18)	1.26 (1.24–1.29)	1.30 (1.28–1.32)	1.14 (1.13–1.15)	1.20 (1.22–1.21)
Traumatic brain injury	1.08 (1.05–1.11)	1.21 (1.17–1.24)	1.33 (1.30–1.36)	1.45 (1.42–1.48)	1.15 (1.13–1.17)	1.27 (1.25–1.29)

HR = hazard ratio; ^a^: Multiple Cox regression was conducted with adjustment for age, urbanicity and low-income status. ^b^: Multiple Cox regression was conducted with adjustment for average parental age, family urbanicity and family low-income status.

## Data Availability

The data presented in this study are available on request from the corresponding author.

## Supplementary Materials

The following are available online at https://www.mdpi.com/article/10.3390/ijerph18073586/s1, Table S1: Reference ICD9/10 code of traumatic injury.
