# Supplementary material for: Increased Risk of Traumatic Injuries among Parents of Children with Attention Deficit/Hyperactivity Disorder: A Nationwide Population-Based Study
Source: Int J Environ Res Public Health. 2021 Mar 30;18(7):3586. doi: 10.3390/ijerph18073586 (PMC8036660; doi:10.3390/ijerph18073586)
Supplement: Supplementary file 1 [file ijerph-18-03586-s001.pdf]

Table S1. Reference ICD9/10 code of traumatic injury.

icd-10			icd-9		
**Burn**	**TBI**	**Fracture**	**Burn**	**TBI**	**Fracture**
T20	S020	S12	940	95901	800
T21	S021	S22	941	850	801
T22	S028	S32	942	851	802
T23	S0291	S42	943	852	803
T24	S0402	S52	944	853	804
T25	S0403	S62	945	854	805
T26	S0404	S72	946		806
T27	S06	S82	947		807
T28	S071	S92	948		808
T29	T744	T02	949		809
T30	S0990	T08			810
T31		T10			811
T32		T12			812
					813
					814
					815
					816
					817
					818
					819
					820
					821
					822
					823
					824
					825
					826
					827
					828
					829
